# Impact of oral health on quality of life among Tibetan adolescents in Suoxian County: a cross-sectional study

**DOI:** 10.3389/fpubh.2026.1814034

**Published:** 2026-08-11

**Authors:** Siyuan Cao, Ying Ji, Qitao Du, Lei Wang, Ma Mi, Xiaopei Du

**Affiliations:** 1 Department of Endodontics, Stomatological Hospital of Dalian University (Dalian Stomatological Hospital), Dalian, China; 2 Department of Pediatric Dentistry, Stomatological Hospital of Dalian University (Dalian Stomatological Hospital), Dalian, China; 3 Department of Comprehensive Department II, Stomatological Hospital of Dalian University (Dalian Stomatological Hospital), Dalian, China; 4 Department of Stomatology, Tibet Autonomous Region Second People's Hospital, Lhasa, China; 5 Suoxian People's Hospital, Nagqu, China

**Keywords:** adolescent, dental caries, gingival hemorrhage, oral health, quality of life

## Abstract

**Background:**

Oral health (OH) is critical to overall wellbeing, yet its impact on quality of life (QoL) among Tibetan adolescents in remote regions such as Suoxian County remains poorly understood. This study investigates the prevalence of oral-health issues and their effects on QoL in this population.

**Methods:**

A cross-sectional study was conducted in Suoxian County, Tibet. The primary cohort comprised 811 adolescents aged 13–15 years recruited from Suo County Middle School, who underwent clinical oral examination and completed the Child Oral Impacts on Daily Performances (Child-OIDP) questionnaire to evaluate oral-health-related quality of life (OHRQoL). A smaller, exploratory adult cohort (*n* = 49) was clinically examined at Suo County People’s Hospital to provide a preliminary descriptive context. However, this cohort did not complete a validated OHRQoL instrument and was not included in the primary analyses. Clinical examinations assessed dental status using the Decayed, Missing, and Filled Teeth (DMFT) index, gingival bleeding, and dental calculus.

**Results:**

Clinical examinations revealed a high prevalence of oral-health problems among Tibetan adolescents (*n* = 811): 48.7% had DMFT >0, 68.9% exhibited gingival bleeding, and 50.3% presented with dental calculus. The Child-OIDP questionnaire demonstrated moderate overall QoL impairment (mean score: 17.6% of maximum), with 75.6% reporting at least one oral impact. Eating was the most affected domain (mean score: 1.1). Regression analyses identified dental calculus (*β* = 0.28, *p* = 0.004) and DMFT >0 (*β* = 0.17, *p* = 0.028) as significant predictors of reduced QoL. Adults (*n* = 49) showed similarly high rates of untreated caries, periodontal disease, and limited dental care access.

**Conclusion:**

Oral-health problems are widespread among Tibetan adolescents and significantly impair their QoL, particularly through effects on eating.

## 1 Introduction

Oral disease is a major health issue across diverse populations (1, 2). Oral health (OH) is a description of the standard of oral health and its related tissues that enables the individual to eat, speak, and socialize without active disease or discomfort, and it contributes to the general wellbeing (3, 4). Oral health is usually assessed using multiple clinical indicators. Clinical indicators alone do not fully explain how oral conditions affect daily activities such as eating, speaking, sleeping, school attendance, and social interaction. Therefore, combining clinical examination with oral-health-related quality of life (OHRQoL) assessment provides a more complete understanding of disease burden (5, 6). So, the assessment of quality of life (QoL) alongside routine clinical assessment is an important health outcome (7). Along with the regular clinical assessments, assessment of quality of life allows healthcare professionals to have the full picture of the treatment protocols and the quality of care from a patient’s perspective. Cross-sectional studies on oral health and its quality-of-life effect have been published in different cultures and countries and have indicated that oral disease and disorders have a negative impact on daily life and wellbeing in school children (8–10). However, evidence remains limited among Tibetan adolescents, particularly in remote areas where access to dental care, oral health literacy, and dietary habits may differ from those of other populations (11, 12). Furthermore, significant methodological limitations in sampling, data collection, and analysis are evident in the published studies on these age groups (13). This gap is important because understanding both the clinical oral-health status and its impact on daily life can help guide school-based prevention, oral-health education, and dental-service planning in remote Tibetan communities. The purpose of this study was to describe oral-health status among Tibetan adolescents in Suoxian County and assess the association between dental problems and quality of life using the Child Oral Impacts on Daily Performances (Child-OIDP) questionnaire.

## 2 Methods

### 2.1 Study design

We conducted cross-sectional surveys in Suoxian County, China. The adolescent survey was conducted from September 2021 to July 2022, and the adult survey was conducted from May to July 2023. The primary cohort comprised school-attending adolescents aged 13–15 years recruited from Suo County Middle School. A smaller exploratory adult cohort was clinically examined at Suo County People’s Hospital during its respective survey period. Data collection was conducted collaboratively by clinicians from the Stomatological Hospital of Dalian University (Dalian Stomatological Hospital) and Suo County People’s Hospital.

### 2.2 Study population inclusion and exclusion criteria

Adolescent participants were eligible if they were aged 12–15 years and enrolled at Suo County Middle School during the study period; all eligible students in this age range were invited through school-based recruitment. Adults included individuals aged 18 years or older seeking routine dental care or accompanying patients at Suo County People’s Hospital. Recruitment was school-based, whole-population sampling for adolescents and convenience sampling for adults (see Section 2.3 “Sample size calculation and method of sampling”).

#### 2.2.1 Exclusion criteria

- Adolescents whose parents or legal guardians did not provide written informed consent.
- Adults who declined to participate.
- Participants with serious systemic conditions (e.g., acute infections, psychiatric disorders) that contraindicated oral examination or interfered with questionnaire completion.

### 2.3 Sample size calculation and method of sampling

The sample size was calculated using OpenEpi version 3.01. Based on pilot data and literature reporting a caries prevalence of ~50% in similar populations, the following parameters were used:

- Expected prevalence: 50% (maximizes variance for conservative estimation)
- Margin of error: ±5%
- Confidence level: 95%
- Design effect: 1.0 (assumed)

The minimum required sample size was 384. To ensure representativeness and enable subgroup analyses, the final sample was increased to 811 adolescents. For the adult cohort, 49 participants were recruited via convenience sampling due to logistical constraints and the exploratory nature of this subgroup; no formal sample size calculation was performed.

For the adolescent cohort, we used a school-based whole-population recruitment approach: every student aged 13–15 years enrolled at Suo County Middle School during the study period was invited to participate. Because only one middle school serves this remote county, this approach effectively constitutes a census of the school-attending adolescent population rather than probability sampling; we therefore did not apply a formal random-selection procedure or replacement sampling. For the adult cohort, convenience sampling was conducted at Suo County People’s Hospital, prioritizing feasibility over representativeness. All participants, or their parents/legal guardians, provided written informed consent. We acknowledge that the adolescent cohort, although large and population based within the school, is geographically limited, and that the adult cohort is convenience based and should be interpreted only as exploratory.

### 2.4 Form of oral assessment

In the first part of our study, we assessed dental status using oral assessments. We used the Decayed, Missing, and Filled Teeth (DMFT) (14). We also examined the caries status for all patients’ teeth, and periodontal assessments included index teeth. Our clinical examination was conducted based on the World Health Organization (WHO) protocol and criteria (single examiner, in natural light, after drying and isolating teeth), using a standard probe and flat mirror. The oral assessment was carried out by a licensed and trained dentist, and we followed standardized protocols.

### 2.5 Child oral impact on daily performance (child-OIDP) questionnaire

We administered the Chinese-language validated version of the Child-OIDP, which was self-completed by the adolescent participants (15, 16). Because our participants were ethnically Tibetan and Tibetan is the dominant household language in Suoxian County, we addressed linguistic and cultural appropriateness through three measures: (i) bilingual Mandarin–Tibetan clinical staff from Suo County People’s Hospital were present during questionnaire administration to read items aloud in Tibetan and clarify wording where requested by participants; (ii) the instrument was pilot-tested with a small group of students prior to data collection to confirm item comprehension; and (iii) because all participants attended Mandarin-medium schooling and were literate in written Chinese, the written instrument was retained while oral Tibetan clarification was offered for every item. We acknowledge that a formal cross-cultural translation and back-translation of the Child-OIDP into Tibetan were not performed; the future studies should undertake full cross-cultural adaptation and psychometric revalidation of the instrument in Tibetan. The first part of the questionnaire is a checklist of oral pathologies and conditions experienced during the past 3 months, intended to orient participants to oral conditions that may affect QoL. The second part assesses the effects of these oral conditions across eight daily performances: (1) eating, (2) speech, (3) oral hygiene, (4) sleeping and resting, (5) emotional state, (6) smiling (showing teeth without embarrassment), (7) study and school attendance, and (8) socializing. For each performance, we recorded both the frequency (0–3) and severity (0–3) of impact; the score for each performance (impact intensity) is their product, ranging from 0 to 9. Total Child-OIDP score was summed across the eight performances and expressed as a percentage of the maximum obtainable score.

### 2.6 Adult questionnaire

Adult participants completed a structured questionnaire based on China’s 4th National Oral Health Survey. We collected information on sociodemographic characteristics (age, gender, ethnicity, and education), lifestyle factors (smoking habits, dietary frequency of sugary items), oral-hygiene practices (brushing frequency, toothpaste/floss use, and fluoride awareness), dental-service utilization (visit history, reasons, and barriers), self-perceived oral and general health status, self-reported oral symptoms, the perceived impact of oral health on life, and basic oral-health knowledge and attitudes.

### 2.7 Statistical data analysis

Descriptive statistics summarized the characteristics of each cohort. Prevalence of clinical outcomes was reported as a count and a percentage. Associations between clinical oral indicators (DMFT >0, gingival bleeding, dental calculus) and Child-OIDP total scores were examined using Spearman’s correlation, linear regression (continuous Child-OIDP), and logistic regression (Child-OIDP >0 vs. =0). All analyses were performed in Jamovi 2.3^1^ and STATA. Statistical significance was set at *p* < 0.05.

### 2.8 Ethical approval

Our study was performed in accordance with the ethical principles of the Declaration of Helsinki. The study protocol was approved by the Biomedical Ethics Committee. Written informed consent was obtained from all adult participants and from the parents or legal guardians of adolescent participants, with assent also obtained from the adolescents.

## 3 Results

Our study population included 811 adolescents aged 13–15 years, all residing in Suoxian County, Tibet. We report demographic characteristics and clinical oral-health findings for each cohort, while OHRQoL results are reported only for adolescents.

### 3.1 Adolescents (13-15 years old)

Our adolescent cohort included 811 participants, with a near-equal gender distribution (49.6% male, *n* = 402; 50.4% female, *n* = 409). Nearly all participants were Tibetan (99.9%, *n* = 810), with only one individual identifying as Chinese (0.1%). The age distribution was heavily skewed toward 15-year old participants (94.2%, *n* = 764), followed by 14-year old participants (4.6%, *n* = 37) and 13-year old participants (1.2%, *n* = 10). Table 1 shows the characteristics of our adolescent cohort.

**Table 1:** Characteristics of our adolescent’s cohort.

Characteristic		Category	Overall (*N* = 811)
Male		402 (49.6%)
Ethnicity	Chinese	1 (0.1%)
	Tibetan	810 (99.9%)
Age group	13-Year old	10 (1.2%)
	14-Year old	37 (4.6%)
	15-Year old	764 (94.2%)

Clinical oral examinations, as summarized in Table 2, showed a high incidence of oral diseases among these age groups. The Decayed, Missing, and Filled Teeth (DMFT) score was greater than zero in 48.7% of participants (*n* = 395), indicating that nearly half of the adolescents had experienced dental caries, tooth loss, or fillings. Gingival bleeding was observed in 68.9% (*n* = 559), and dental calculus was present in 50.3% (*n* = 408), suggesting widespread periodontal issues.

**Table 2:** Clinical findings and oral examination results.

Tooth ID	Dental crown condition		Gingival bleeding in each tooth	Calculus formation in each tooth
11	Bridge abutment tooth, special crown or veneer	1 (0.1%)	50 (6.2%)	31 (3.8%)
	Crowned teeth	7 (0.9%)		
	Missing due to other reasons	2 (0.2%)		
	No teeth	800 (98.6%)		
	Trauma group	1 (0.1%)		
12	Crowned teeth	2 (0.2%)	50 (6.2%)	32 (3.9%)
	Missing due to tooth loss	1 (0.1%)		
	No teeth	806 (99.4%)		
	Unerupted teeth	2 (0.2%)		
13	Crowned teeth	3 (0.4%)	49 (6.0%)	31 (3.8%)
	Missing due to other reasons	1 (0.1%)		
	No teeth	806 (99.4%)		
	Unerupted teeth	1 (0.1%)		
14	Crowned teeth	10 (1.2%)	22 (2.7%)	11 (1.4%)
	No teeth	800 (98.6%)		
	Unerupted teeth	1 (0.1%)		
15	Crowned teeth	6 (0.7%)	21 (2.6%)	6 (0.7%)
	No teeth	803 (99.0%)		
	Unerupted teeth	2 (0.2%)		
16	Crowned teeth	53 (6.5%)	150 (18.5%)	54 (6.7%)
	Missing due to other reasons	2 (0.2%)		
	Missing due to tooth loss	5 (0.6%)		
	Filled teeth	2 (0.2%)		
	No teeth	749 (92.4%)		
17	Crowned teeth	42 (5.2%)	150 (18.5%)	53 (6.5%)
	Missing due to other reasons	4 (0.5%)		
	No teeth	760 (93.7%)		
	Unerupted teeth	5 (0.6%)		
21	Crowned teeth	4 (0.5%)	45 (5.5%)	29 (3.6%)
	Missing due to other reasons	1 (0.1%)		
	No teeth	805 (99.3%)		
	Trauma group	1 (0.1%)		
22	Crowned teeth	2 (0.2%)	45 (5.5%)	27 (3.3%)
	No teeth	805 (99.3%)		
	Unerupted teeth	4 (0.5%)		
23	Crowned teeth	3 (0.4%)	44 (5.4%)	26 (3.2%)
	No teeth	808 (99.6%)		
24	Crowned teeth	9 (1.1%)	19 (2.3%)	9 (1.1%)
	Missing due to other reasons	1 (0.1%)		
	No teeth	798 (98.4%)		
	Unerupted teeth	3 (0.4%)		
25	Crowned teeth	9 (1.1%)	17 (2.1%)	6 (0.7%)
	Missing due to tooth loss	1 (0.1%)		
	No teeth	800 (98.6%)		
	Unerupted teeth	1 (0.1%)		
26	Crowned teeth	74 (9.1%)	142 (17.5%)	61 (7.5%)
	Missing due to other reasons	2 (0.2%)		
	Missing due to tooth loss	3 (0.4%)		
	Filled but missing teeth	1 (0.1%)		
	No teeth	731 (90.1%)		
27	Crowned teeth	40 (4.9%)	142 (17.5%)	60 (7.4%)
	Missing due to other reasons	5 (0.6%)		
	Missing due to tooth loss	1 (0.1%)		
	No teeth	758 (93.5%)		
	Unerupted teeth	7 (0.9%)		
31	Missing due to other reasons	1 (0.1%)	190 (23.4%)	162 (20.0%)
	No teeth	810 (99.9%)		
32	No teeth	810 (99.9%)	220 (27.1%)	188 (23.2%)
	Unerupted teeth	1 (0.1%)		
33	Missing due to other reasons	1 (0.1%)	150 (18.5%)	144 (17.8%)
	No teeth	809 (99.8%)		
	Not recorded	1 (0.1%)		
34	Crowned teeth	3 (0.4%)	12 (1.5%)	9 (1.1%)
	No teeth	807 (99.5%)		
	Unerupted teeth	1 (0.1%)		
35	Crowned teeth	10 (1.2%)	22 (2.7%)	11 (1.4%)
	Missing due to other reasons	1 (0.1%)		
	Missing due to tooth loss	4 (0.5%)		
	No teeth	795 (98.0%)		
	Unerupted teeth	1 (0.1%)		
36	Crowned teeth	138 (17.0%)	49 (6.0%)	19 (2.3%)
	Missing due to other reasons	6 (0.7%)		
	Missing due to tooth loss	7 (0.9%)		
	No teeth	660 (81.4%)		
37	Crowned teeth	107 (13.2%)	45 (5.5%)	18 (2.2%)
	Missing due to tooth loss	1 (0.1%)		
	No teeth	701 (86.4%)		
	Unerupted teeth	2 (0.2%)		
41	Crowned teeth	1 (0.1%)	203 (25.0%)	176 (21.7%)
	Missing due to other reasons	1 (0.1%)		
	No teeth	809 (99.8%)		
42	No teeth	811 (100.0%)	244 (30.1%)	214 (26.4%)
43	No teeth	809 (99.8%)	156 (19.2%)	150 (18.5%)
	Not recorded	1 (0.1%)		
	Unerupted teeth	1 (0.1%)		
44	Crowned teeth	1 (0.1%)	13 (1.6%)	10 (1.2%)
	No teeth	809 (99.8%)		
	Unerupted teeth	1 (0.1%)		
45	Crowned teeth	11 (1.3%)	15 (1.8%)	11 (1.4%)
	Missing due to other reasons	4 (0.5%)		
	Missing due to tooth loss	2 (0.2%)		
	No teeth	792 (97.7%)		
	Unerupted teeth	2 (0.2%)		
46	Crowned teeth	144 (17.8%)	51 (6.3%)	15 (1.8%)
	Missing due to other reasons	12 (1.5%)		
	Missing due to tooth loss	16 (2.0%)		
	Filled teeth	2 (0.2%)		
	No teeth	637 (78.5%)		
47	Crowned teeth	97 (12.0%)	50 (6.2%)	15 (1.9%)
	Missing due to other reasons	1 (0.1%)		
	No teeth	711 (87.7%)		
	Unerupted teeth	2 (0.2%)		
Decayed, Missing, and Filled Teeth (DMFT) score> 0		395 (48.7%)		
Total participants who have gingival bleeding		559 (68.9%)		
The total number of participants who have dental calculus		408 (50.3%)		

Tooth-specific findings further stress the prevalence of oral-health problems. For instance, tooth 36 (first mandibular molar) exhibited crowned status in 17% of cases (*n* = 138), while tooth 46 (its contralateral counterpart) showed a similar pattern (17.8%, *n* = 144). Gingival bleeding and calculus formation were notably higher in the mandibular anterior teeth, with tooth 42 showing bleeding in 30.1% (*n* = 244) and calculus in 26.4% (*n* = 214) of participants.

OHRQoL was assessed using the Child-OIDP questionnaire—the mean total score was 4.2 [standard deviation (SD) = 4.6]. When normalized to a percentage of the maximum possible score, the mean Child-OIDP score was 17.6% (SD = 19.3%), indicating a moderate overall impact of oral health on daily life. Notably, 75.6% of adolescents (*n* = 613) reported at least one oral impact (Child-OIDP > 0). Quality-of-life results are summarized in Tables 3, 4.

**Table 3:** Summary of quality-of-life assessment results for 13–15-year-old adolescents.

Outcome	Mean (SD), or *N* (%)
Child-OIDP total score	4.2 (4.6)
Child-OIDP total score/24	0.2 (0.2)
Child-OIDP total score/24 × 100	17.6 (19.3)
Number of participants with Child-OIDP > 0	613 (75.6%)
Dental filling	5 (0.6%)
Child-OIDP eating	1.1 (1.0)
Child-OIDP pronunciation	0.5 (0.8)
Child-OIDP brushing or rinsing	0.5 (0.9)
Child-OIDP attending school	0.4 (0.8)
Child-OIDP sleeping	0.5 (0.9)
Child-OIDP smiling with teeth	0.5 (0.9)
Child-OIDP mood and emotions	0.5 (0.9)
Child-OIDP making friends	0.2 (0.7)
Child-OIDP impact level	1.5 (1.1)

**Table 4:** Survey results for 13–15-year-old adolescents.

Survey item	Response category	*N* (%) or mean (SD)
Only child or not	1: Only child	49 (6.0%)
	2: Not unique	762 (94.0%)
Father’s education level	1: No education	527 (65.0%)
	2: Compulsory education	175 (21.6%)
	3. Higher education	17 (2.1%)
	4: Unknown or orphan	92 (11.3%)
Mother’s education level	1: No education	623 (76.8%)
	2: Compulsory education	100 (12.3%)
	3. Higher education	12 (1.5%)
	4: Unknown or orphan	76 (9.4%)
Brushing frequency	1: ≥ 2 times per day	76 (9.4%)
	2: Once a day	633 (78.1%)
	3: Not brushing every day	102 (12.6%)
Use of fluoride toothpaste	1: Yes	313 (38.6%)
	2: No	71 (8.8%)
	3: I do not know	427 (52.7%)
Use of dental floss	1: Frequently used	23 (2.8%)
	2: Occasionally use	381 (47.0%)
	3: No	407 (50.2%)
Sweet snacks	1: ≤ 1 time per week	218 (26.9%)
	2: 2–6 times a week	267 (32.9%)
	3: ≥1 time per day	326 (40.2%)
Sweet beverages	1: ≤ 1 time per week	293 (36.1%)
	2: 2–6 times a week	296 (36.5%)
	3: ≥1 time per day	222 (27.4%)
Sweetened dairy products	1: ≤ 1 time per week	374 (46.1%)
	2: 2–6 times a week	222 (27.4%)
	3: ≥1 time per day	215 (26.5%)
Smoking	1: Never	623 (76.8%)
	2: Rarely or once	118 (14.5%)
	3: Weekly	22 (2.7%)
	4: Every day	48 (5.9%)
Self-rated oral health	1: Good or very good	397 (49.0%)
	2: General	326 (40.2%)
	3: Poor or very poor	88 (10.9%)
Dental trauma	1: No injury	645 (79.5%)
	2: Injured	166 (20.5%)
Toothache in the last 12 months	1: Never	329 (40.6%)
	2: Occasionally	425 (52.4%)
	3: Often	57 (7.0%)
Visited a medical institution for dental care	1: Viewed	185 (22.8%)
	2: Never seen it	626 (77.2%)
Mean oral-health knowledge score	4.0 (2.1)	
Oral-health knowledge level	1: Poor knowledge level	197 (24.3%)
	2: Medium	386 (47.6%)
	3: Good	228 (28.1%)
Mean oral-health attitude score	3.4 (0.9)	
Oral-health attitude level	1. Negative attitude	106 (13.1%)	
	2: Medium	199 (24.5%)	
	3. Positive attitude	506 (62.4%)
Test calculation: dental trauma	Yes, injured	166 (20.5%)
	No, never injured	645 (79.5%)
Test calculation: toothache in last 12 months	Often	57 (7.0%)
	Occasionally	425 (52.4%)
	Never	329 (40.6%)

Among the eight daily performances evaluated, eating was the most affected, with a mean score of 1.1 (SD = 1), followed by pronunciation, brushing or rinsing, sleeping, smiling, mood and emotions (all with mean scores of 0.5, SD ranging from 0.8 to 0.9), attending school (mean = 0.4, SD = 0.8), and making friends (mean = 0.2, SD = 0.7). The mean impact level across these domains was 1.5 (SD = 1.1), reflecting a consistent yet modest influence of oral conditions on daily functioning.

Survey responses provided additional context for these clinical and QoL findings. The majority of the adolescents (94%, *n* = 762) were not the only children, and parental education levels were generally low, with 65% of fathers (*n* = 527) and 76.8% of mothers (*n* = 623) reporting no formal education. Oral-hygiene practices were suboptimal: only 9.4% (*n* = 76) brushed twice daily, 78.1% (*n* = 633) brushed once daily, and 12.6% (*n* = 102) did not brush every day. Use of fluoride toothpaste was reported by 38.6% (*n* = 313), but 52.7% (*n* = 427) were unsure if their toothpaste contained fluoride. Dental floss use was rare, with 50.2% (*n* = 407) reporting they never use it. Dietary habits showed frequent consumption of sweets, with 40.2% (*n* = 326) eating sweet snacks daily and 27.4% (*n* = 222) consuming sweet beverages daily.

Smoking was not common, with 76.8% (*n* = 623) never smoking, though 5.9% (*n* = 48) smoked daily. Self-rated oral health was good or very good in 49% (*n* = 397), but a significant portion reported a toothache. When we asked about toothache frequency in the past 12 months, 57 adolescents (7%) reported experiencing it often, 425 (52.4%) occasionally, and 329 (40.6%) never. Regarding self-reported dental trauma, 645 participants (79.5%) reported having experienced an injury to their teeth, while 166 (20.5%) reported no such injury. Also, 77.2% (*n* = 626) had never visited a dental care facility. Oral-health knowledge and attitudes were moderate, with mean scores of 4 (SD = 2.1) and 3.4 (SD = 0.9), respectively.

Regression analyses were performed to examine associations between clinical oral-health indicators and Child-OIDP scores. The presence of dental calculus was significantly associated with higher Child-OIDP scores [estimate = 1.28, standard error (SE) = 0.44, *p* = 0.004], suggesting a stronger impact on QoL (standardized estimate = 0.28, 95% CI: 0.09–0.46). The detailed results of the regression models are reported in Table 5.

**Table 5:** Regression models to correlate the total Child-OIDP score with DMFT, dental calculus, and gingival bleeding as a factor in child participants.

Model coefficients—Child-OIDP total score									
Predictor	Estimate	SE	95% Confidence interval		*t*	*p*	Standard estimate	95% Confidence interval	
			Lower	Upper				Lower	Upper
Intercept^a^	3.865	0.296	3.2838	4.4467	13.05	< 0.001			
Gingival bleeding									
2: Bleeding gums – 1: No bleeding gums	−0.989	0.510	−1.9908	0.0127	−1.94	0.053	−0.214	−0.4300	0.00275
DMFT									
2: DMFT>0–1: DMFT = 0	0.789	0.358	0.0862	1.4928	2.20	0.028	0.171	0.0186	0.32241
Dental Calculus									
2: There is dental calculus – 1: No Calculus	1.283	0.440	0.4194	2.1457	2.92	0.004	0.277	0.0906	0.46342
^a^Represents reference level.									

Model coefficients—Child-OIDP whether > 0									
Predictor	Estimate	95% Confidence interval		SE	*Z*	*p*	Odds ratio	95% Confidence interval	
		Lower	Upper					Lower	Upper
Intercept	1.156	0.8574	1.454	0.152	7.59	< 0.001	3.177	2.357	4.282
DMFT									
2: DMFT>0–1: DMFT = 0	0.326	−0.0392	0.691	0.186	1.75	0.080	1.385	0.962	1.996
Gingival bleeding									
2: Bleeding gums – 1: No bleeding gums	−1.021	−1.5028	−0.540	0.246	−4.16	< 0.001	0.360	0.223	0.583
Dental calculus									
2: There is dental calculus – 1: No calculus	1.119	0.7077	1.531	0.210	5.33	< 0.001	3.063	2.029	4.622

Estimates represent the log odds of “Child-OIDP Whether > 0 = Child-OIDP>0” vs. “Child-OIDP Whether > 0 = Child-OIDP = 0”.

**Figure 1:**
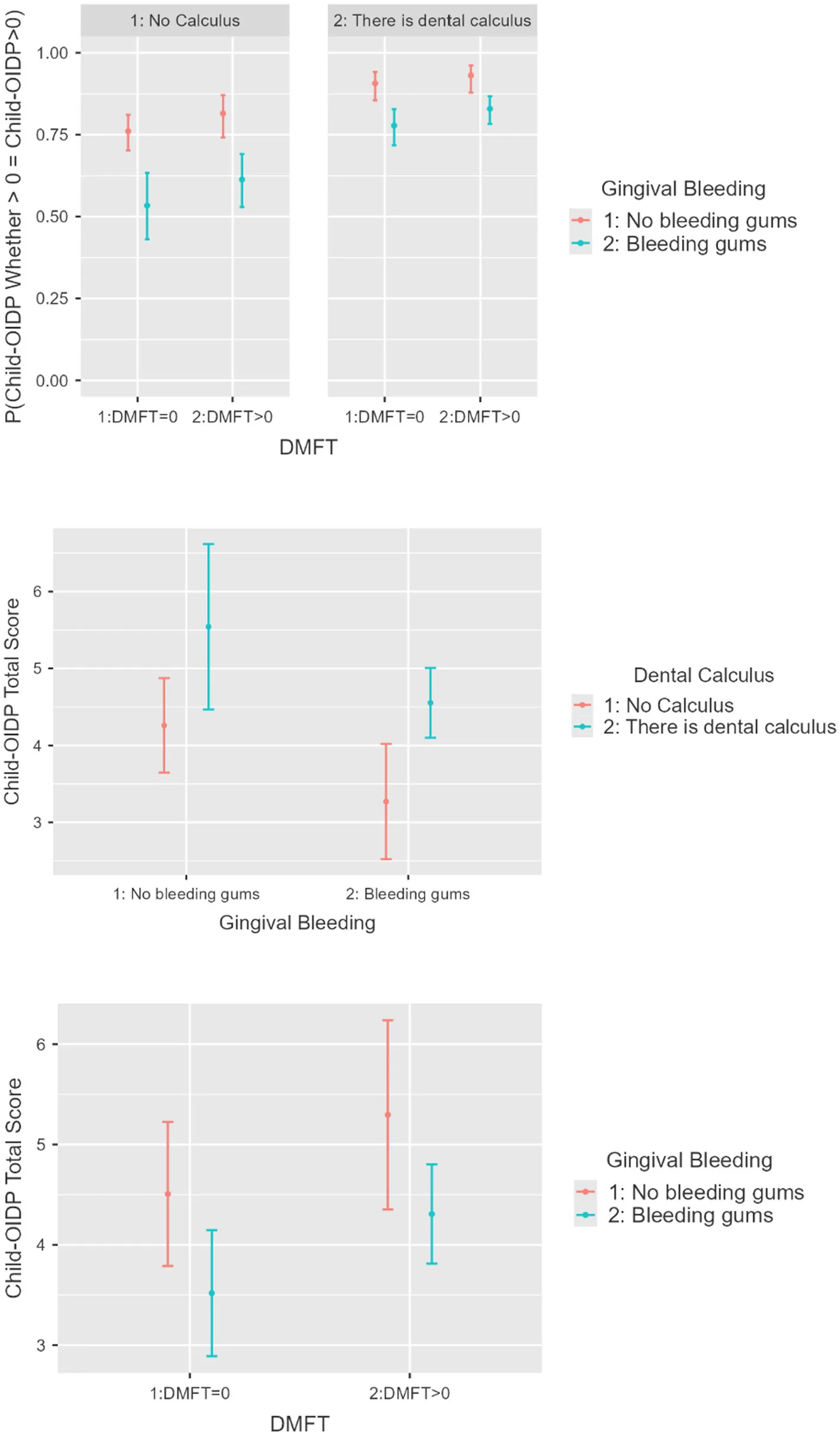
Marginal model estimates (with error bars) showing associations of DMFT, gingival bleeding, and dental calculus with (top) the probability of any Child-OIDP impact and (middle and bottom) the Child-OIDP total score.

DMFT score greater than zero also correlated with increased Child-OIDP scores (estimate = 0.789, SE = 0.358, *p* = 0.028; standardized estimate = 0.17, 95% CI: 0.019–0.32), while gingival bleeding showed a non-significant negative association (estimate = − 0.99, SE = 0.51, *p* = 0.053). Logistic regression further indicated that dental calculus significantly increased the odds of reporting any oral impact on quality of life (OR = 3.06, 95% CI: 2.03–4.62, *p* < 0.001), whereas gingival bleeding reduced the odds (OR = 0.36, 95% CI: 0.22–0.58, p < 0.001), and DMFT > 0 showed a non-significant trend (OR = 1.39, 95% CI: 0.96–1.996, *p* = 0.08). These findings underscore the significant role of dental caries and calculus in impairing QoL among Tibetan adolescents (see Figure 1). 

The characteristics, clinical findings, questionnaire results, and denture restoration status of the exploratory adult cohort are presented in Tables 6–9, respectively.

**Table 6:** Characteristics of adult participants.

Characteristic		Category	Overall (*N* = 49)
Male		20 (40.8%)
Ethnicity	Tibetan	49 (100%)
Age		43.2 (9.2)
*Age groups*	≤ 45 years	46 (93.9%)
	> 45 years	3 (6.1%)

**Table 7:** Clinical findings and oral examination results for adults.

Tooth ID	Dental crown condition		Dental root condition		Gingival bleeding	Calculus formation	Periodontal pocket		Periodontal attachment loss
11	No caries	45 (91.8%)	No caries	4 (8.2%)	27 (55.1%)	21 (42.9%)	One pocket	13 (26.5%)	Loss: 13 (26.5%)
	Crown caries	3 (6.1%)	Unerupted	44 (89.8%)					
							Two pockets	0	
	Missing due to other reasons	1 (2.0%)	NR	1 (2.0%)					
12	No caries	45 (91.8%)	No caries	2 (4.1%)	25 (51.0%)	19 (38.8%)	One pocket	7 (14.3%)	–
	Crown caries	2 (4.1%)	Unerupted	46 (93.9%)					
	Missing due to other reasons	1 (2.0%)	NR	1 (2.0%)			Two pockets	1 (2.0%)	
	Bridge abutment, special crown/veneer	1 (2.0%)							
13	No caries	48 (98.0%)	No caries	3 (6.1%)	25 (51.0%)	19 (38.8%)	One pocket	6 (12.2%)	–
	Missing due to other reasons	1 (2.0%)	Unerupted	45 (91.8%)					
							Two pockets		
			NR	1 (2.0%)					
14	No caries	45 (91.8%)	No caries	4 (8.2%)	20 (40.8%)	17 (34.7%)	One pocket	5 (10.2%)	–
	Crown caries	3 (6.1%)	Unerupted	1 (2.0%)					
	Missing due to other reasons	1 (2.0%)	Pit and fissure sealant	43 (87.8%)			Two pockets		
			NR	1 (2.0%)					
15	No caries	39 (79.6%)	No caries	4 (8.2%)	22 (44.9%)	19 (38.8%)	One pocket	5 (10.2%)	–
	Crown caries	8 (16.3%)	Crown caries	1 (2.0%)					
	Missing due to other reasons	2 (4.1%)	Pit and fissure sealant	1 (2.0%)					
							Two pockets		
			Unerupted	42 (85.7%)					
			NR	1 (2.0%)					
16	No caries	32 (65.3%)	No caries	7 (14.3%)	24 (49.0%)	22 (44.9%)	One pocket	11 (22.4%)	16/17 Loss: 13 (26.5%)
	Crown caries	13 (26.5%)	Crown caries	2 (4.1%)					
	Filled with caries	1 (2.0%)	Unerupted	37 (75.5%)			Two pockets	5 (10.2%)	
	Missing due to other reasons	3 (6.1%)	NR	3 (6.1%)					
17	No caries	30 (61.2%)	No caries	3 (6.1%)	22 (44.9%)	18 (36.7%)	One pocket	9 (18.4%)	–
	Crown caries	14 (28.6%)	Crown caries	2 (4.1%)					
	Missing due to other reasons	5 (10.2%)	Pit and fissure sealant	1 (2.0%)					
							Two pockets	4 (8.2%)	
			Unerupted	37 (75.5%)					
			NR	6 (12.2%)					
18	No caries	27 (55.1%)	No caries	3 (6.1%)	21 (42.9%)	20 (40.8%)	One pocket	9 (18.4%)	–
	Crown caries	14 (28.6%)	Crown caries	1 (2.0%)			Two pockets	1 (2.0%)	
	Missing due to other reasons	4 (8.2%)	Pit and fissure sealant	2 (4.1%)					
	Pit and fissure sealant	1 (2.0%)	Unerupted	37 (75.5%)					
	Unerupted	3 (6.1%)	NR	6 (12.2%)					
21	No caries	45 (91.8%)	No caries	4 (8.2%)	27 (55.1%)	19 (38.8%)	One pocket	10 (20.4%)	–
	Crown caries	3 (6.1%)	Unerupted	44 (89.8%)					
							Two pockets	1 (2.0%)	
	Missing due to other reasons	1 (2.0%)	NR	1 (2.0%)					
22	No caries	46 (93.9%)	No caries	3 (6.1%)	25 (51.0%)	19 (38.8%)	One pocket	8 (16.3%)	–
	Crown caries	2 (4.1%)	Unerupted	45 (91.8%)					
							Two pockets		
	Missing due to other reasons	1 (2.0%)	NR	1 (2.0%)					
23	No caries	49 (100.0%)	No caries	3 (6.1%)	26 (53.1%)	19 (38.8%)	One pocket	6 (12.2%)	–
			Unerupted	46 (93.9%)			Two pockets		
24	No caries	48 (98.0%)	No caries	3 (6.1%)	22 (44.9%)	17 (34.7%)	One pocket	5 (10.2%)	–
	Crown caries	1 (2.0%)	Crown caries	1 (2.0%)					
							Two pockets		
			Unerupted	45 (91.8%)					
25	No caries	45 (91.8%)	No caries	2 (4.1%)	21 (42.9%)	18 (36.7%)	One pocket	4 (8.2%)	–
	Crown caries	1 (2.0%)	Pit and fissure sealant	1 (2.0%)					
							Two pockets		
	Missing due to other reasons	3 (6.1%)	Unerupted	43 (87.8%)					
			NR	3 (6.1%)					
26	No caries	32 (65.3%)	No caries	6 (12.2%)	25 (51.0%)	21 (42.9%)	One pocket	10 (20.4%)	Loss: 13 (26.5%)
	Crown caries	13 (26.5%)	Crown caries	1 (2.0%)					
	Missing due to other reasons	4 (8.2%)	Pit and fissure sealant	1 (2.0%)					
							Two pockets	2 (4.1%)	
			Unerupted	37 (75.5%)					
			NR	4 (8.2%)					
27	No caries	30 (61.2%)	No caries	4 (8.2%)	22 (44.9%)	19 (38.8%)	One pocket	12 (24.5%)	
	Crown caries	12 (24.5%)	Crown caries	1 (2.0%)					
	Missing due to other reasons	7 (14.3%)	Pit and fissure sealant	1 (2.0%)					
							Two pockets	1 (2.0%)	
			Unerupted	35 (71.4%)					
			NR	8 (16.3%)					
28	No caries	31 (63.3%)	No caries	2 (4.1%)	23 (46.9%)	18 (36.7%)	One pocket	8 (16.3%)	–
	Crown caries	11 (22.4%)	Pit and fissure sealant	1 (2.0%)					
	Missing due to other reasons	6 (12.2%)	Unerupted teeth	42 (85.7%)			Two pockets	1 (2.0%)	
	Unerupted	1 (2.0%)	Unerupted	4 (8.2%)					
31	No caries	43 (87.8%)	No caries	4 (8.2%)	40 (81.6%)	39 (79.6%)	One pocket	19 (38.8%)	Loss: 22 (44.9%)
	Crown caries	2 (4.1%)	Crown caries	1 (2.0%)					
	Missing due to other reasons	3 (6.1%)	Unerupted	41 (83.7%)					
	Bridge abutment, special crown/veneer	1 (2.0%)	NR	3 (6.1%)			Two pockets	1 (2.0%)	
32	No caries	47 (95.9%)	No caries	8 (16.3%)	43 (87.8%)	42 (85.7%)	One pocket	17 (34.7%)	–
	Crown caries	1 (2.0%)	Crown caries	1 (2.0%)					
							Two pockets	1 (2.0%)	
	Bridge abutment tooth, special crown or veneer	1 (2.0%)	Unerupted	40 (81.6%)					
33	No caries	47 (95.9%)	No caries	4 (8.2%)	37 (75.5%)	37 (75.5%)	One pocket	10 (20.4%)	–
	Crown caries	1 (2.0%)	Unerupted	45 (91.8%)			Two POCKETS		
	Bridge abutment tooth, special crown or veneer	1 (2.0%)							
34	No caries	47 (95.9%)	No caries	2 (4.1%)	23 (46.9%)	19 (38.8%)	One pocket	6 (12.2%)	–
	Crown caries	1 (2.0%)	Unerupted	47 (95.9%)					
							Two pockets	1 (2.0%)	
	Missing due to other reasons	1 (2.0%)							
35	No caries	42 (85.7%)	No caries	2 (4.1%)	20 (40.8%)	19 (38.8%)	One pocket	5 (10.2%)	–
	Crown caries	4 (8.2%)	Unerupted	45 (91.8%)					
							Two pockets	1 (2.0%)	
	Missing due to other reasons	3 (6.1%)	NR	2 (4.1%)					
36	No caries	27 (55.1%)	No caries	4 (8.2%)	20 (40.8%)	21 (42.9%)	One pocket	9 (18.4%)	Loss: 12 (24.5%)
	Crown caries	14 (28.6%)	Crown caries	1 (2.0%)					
							Two pockets	3 (6.1%)	
	Filled without caries	1 (2.0%)	Unerupted	35 (71.4%)					
	Missing due to other reasons	7 (14.3%)	NR	9 (18.4%)					
37	No caries	22 (44.9%)	No caries	3 (6.1%)	18 (36.7%)	17 (34.7%)	One pocket	8 (16.3%)	–
	Crown caries	16 (32.7%)	Pit and fissure sealant	1 (2.0%)					
	Filled with caries	1 (2.0%)	Unerupted	37 (75.5%)			Two pockets	1 (2.0%)	
	Missing due to other reasons	10 (20.4%)	NR	8 (16.3%)					
38	No caries	33 (67.3%)	No caries	2 (4.1%)	21 (42.9%)	20 (40.8%)	One pocket	8 (16.3%)	–
	Crown caries	11 (22.4%)	Pit and fissure sealant	1 (2.0%)					
	Missing due to other reasons	2 (4.1%)	Unerupted	43 (87.8%)			Two pockets	2 (4.1%)	
	Unerupted	3 (6.1%)	NR	3 (6.1%)					
41	No caries	44 (89.8%)	No caries	7 (14.3%)	41 (83.7%)	40 (81.6%)	One pocket	20 (40.8%)	–
	Crown caries	2 (4.1%)	Crowned caries	1 (2.0%)					
							Two pockets	1 (2.0%)	
	Missing due to other reasons	2 (4.1%)	Unerupted	39 (79.6%)					
	Bridge abutment tooth, special crown or veneer	1 (2.0%)	NR	2 (4.1%)					
42	No caries	47 (95.9%)	No caries	6 (12.2%)	42 (85.7%)	42 (85.7%)	One pocket	16 (32.7%)	–
	Crown caries	1 (2.0%)	Crowned caries	1 (2.0%)					
							Two pockets	1 (2.0%)	
	Bridge abutment tooth, special crown or veneer	1 (2.0%)	Unerupted	42 (85.7%)					
43	No caries	48 (98.0%)	No caries	4 (8.2%)	37 (75.5%)	35 (71.4%)	One pocket	8 (16.3%)	–
	Bridge abutment tooth, special crown or veneer	1 (2.0%)	Unerupted	45 (91.8%)			Two pockets		
44	No caries	48 (98.0%)	No caries	2 (4.1%)	22 (44.9%)	22 (44.9%)	One pocket	6 (12.2%)	–
	Missing due to other reasons	1 (2.0%)	Unerupted	45 (91.8%)					
							Two pockets	1 (2.0%)	
			NR	2 (4.1%)					
45	No caries	42 (85.7%)	No caries	1 (2.0%)	18 (36.7%)	17 (34.7%)	One pocket	5 (10.2%)	–
	Crown caries	1 (2.0%)	Crowned caries	1 (2.0%)					
	Missing due to other reasons	5 (10.2%)	Unerupted	43 (87.8%)			Two pockets	1 (2.0%)	
	Bridge abutment tooth, special crown or veneer	1 (2.0%)	NR	4 (8.2%)					
46	No caries	33 (67.3%)	No caries	3 (6.1%)	18 (36.7%)	18 (36.7%)	One pocket	8 (16.3%)	46/47 Loss: 12 (24.5%)
	Crown caries	7 (14.3%)	Crowned caries	3 (6.1%)					
	Missing due to other reasons	8 (16.3%)	Unerupted	36 (73.5%)			Two pockets	3 (6.1%)	
	Bridge abutment tooth, special crown or veneer	1 (2.0%)	NR	7 (14.3%)					
47	No caries	33 (67.3%)	No caries	5 (10.2%)	20 (40.8%)	18 (36.7%)	One pocket	10 (20.4%)	
	Crown caries	10 (20.4%)	Crowned caries	1 (2.0%)					
	Missing due to other reasons	4 (8.2%)	Unerupted	38 (77.6%)			Two pockets	2 (4.1%)	
	Bridge abutment tooth, special crown or veneer	2 (4.1%)	NR	5 (10.2%)					
48	No caries	37 (75.5%)	No caries	2 (4.1%)	23 (46.9%)	20 (40.8%)	One pocket	9 (18.4%)	–
	Crown caries	9 (18.4%)							
	Missing due to other reasons	2 (4.1%)	Pit and fissure sealant	1 (2.0%)					
							Two pockets	2 (4.1%)	
	Unerupted	1 (2.0%)	Unerupted	46 (93.9%)					

**Table 8:** Questionnaire results for the adult participants.

Questionnaire item/Response category		Overall (*N* = 49)
Highest education level		
No education		43 (87.8%)
Compulsory education		2 (4.1%)
Higher Education		3 (6.1%)
Unknown or Orphan		1 (2.0%)
Sweet snacks		
Rarely/Never		42 (85.7%)
Every week, 2–6 times		2 (4.1%)
Every day, 1 time		3 (6.1%)
Every day, ≥ 2 times		2 (4.1%)
Sweet beverages		
Rarely/Never		46 (93.9%)
Every week, 1 time		1 (2.0%)
Every week, 2–6 times		1 (2.0%)
Every day, ≥ 2 times		1 (2.0%)
Sweetened dairy products		
Rarely/Never		35 (71.4%)
Every week, 1 time		1 (2.0%)
Every week, 2–6 times		1 (2.0%)
Every day, 1 time		11 (22.4%)
Every day, ≥ 2 times		1 (2.0%)
Smoking		
Rarely/Never		4 (8.2%)
Every month, 1–3 times		43 (87.8%)
Every week, 1 time		2 (4.1%)
Years of smoking		
N-Miss		45
Every month, 1–3 times		1 (25.0%)
Every day, 1 time		2 (50.0%)
N		1 (25.0%)
Average daily smoking		
N-Miss		46
Rarely/Never		1 (33.3%)
Every month, 1–3 times		1 (33.3%)
Every week, 1 time		1 (33.3%)
Brushing		
Rarely/Never		10 (20.4%)
Every month, 1–3 times		3 (6.1%)
Every week, 1 time		5 (10.2%)
Every week, 2–6 times		2 (4.1%)
Every day, 1 time		23 (46.9%)
Every day, ≥ 2 times		6 (12.2%)
Toothpick		
Rarely/Never		33 (67.3%)
Every month, 1–3 times		3 (6.1%)
Every week, 1 time		5 (10.2%)
Every day, 1 time		7 (14.3%)
Every day, ≥ 2 times		1 (2.0%)
Dental floss		
Rarely/Never		37 (75.5%)
Every month, 1–3 times		2 (4.1%)
Every week, 1 time		5 (10.2%)
Every day, 1 time		4 (8.2%)
Every day, ≥ 2 times		1 (2.0%)
Use of toothpaste		
Rarely/Never		37 (75.5%)
Every month, 1–3 times		12 (24.5%)
Fluoride toothpaste		
Rarely/Never		2 (4.1%)
Every month, 1–3 times		28 (57.1%)
Every week, 1 time		19 (38.8%)
Have you visited a dentist?		
Rarely/Never		8 (16.3%)
Every month, 1–3 times		41 (83.7%)
Time since last dental visit		
N-Miss		22
Rarely/Never		4 (14.8%)
Every week, 1 time		23 (85.2%)
Reason for dental visit		
N-Miss		27
Rarely/Never		7 (31.8%)
Every week, 1 time		9 (40.9%)
Every week, 2–6 times		6 (27.3%)
Cost of dental visit		
N-Miss		27
0		14 (63.6%)
100		1 (4.5%)
1,000		2 (9.1%)
200		2 (9.1%)
N		3 (13.6%)
No dental problems		
N-Miss		24
Rarely/Never		25 (100.0%)
Dental problems not severe		
N-Miss		39
Rarely/Never		8 (80.0%)
Every month, 1–3 times		2 (20.0%)
No time		
N-Miss		22
Rarely/Never		27 (100.0%)
Financial difficulties		
N-Miss		45
Rarely/Never		4 (100.0%)
No reimbursement		
N-Miss		31
Rarely/Never		18 (100.0%)
No dentist available		
N-Miss		27
Rarely/Never		22 (100.0%)
Fear of dental visits		
N-Miss		36
Rarely/Never		13 (100.0%)
Difficulty finding a dentist		
N-Miss		45
Rarely / Never		4 (100.0%)
Have you had a teeth cleaning?		
Rarely / Never		1 (2.0%)
Every month, 1–3 times		48 (98.0%)
Oral problems affecting life		
Rarely/Never		2 (4.1%)
Every month, 1–3 times		34 (69.4%)
Every week, 1 time		12 (24.5%)
Every week, 2–6 times		1 (2.0%)
Overall health evaluation		
Every month, 1-– times		44 (89.8%)
Every week, 1 time		5 (10.2%)
Teeth and oral evaluation		
Every month, 1–3 times		46 (93.9%)
Every week, 1 time		3 (6.1%)
Oral health is important		
Rarely/Never		49 (100.0%)
Oral-health education level	Regular check-ups are necessary	49 (100.0%)
	Teeth quality is genetic	41 (83.7%)
	Preventing dental diseases depends on me	49 (100.0%)
	Bleeding while brushing is normal	49 (100.0%)
	Bacteria cause gum inflammation	47 (95.9%)
	Brushing has no preventive effect	39 (79.6%)
	Bacteria cause tooth decay	46 (93.9%)
	Eating sugar causes tooth decay	45 (91.8%)
	Fluoride has no effect on teeth	6 (12.2%)
	Pit and fissure sealants protect teeth	47 (95.9%)
	Oral diseases affect overall health	49 (100.0%)

**Table 9:** Denture restoration status for our included adult population.

Denture restoration status	Overall (*N* = 49)
Maxillary—implant	0 (0%)
Maxillary—fixed	0 (0%)
Maxillary—removable	0 (0%)
Maxillary—complete denture	0 (0%)
Maxillary—non-standard	1 (2.0%)
Maxillary—unrestored	18 (36.7%)
Mandibular—implant	0 (0%)
Mandibular—fixed	1 (2.0%)
Mandibular—removable	0 (0%)
Mandibular—complete denture	0 (0%)
Mandibular—non-standard	2 (4.1%)
Mandibular—unrestored	19 (38.8%)

## 4 Discussion

Three findings emerge from our study. First, oral disease is highly prevalent in school-aged Tibetan adolescents in Suoxian County, with roughly half showing caries experience (DMFT >0) and half showing dental calculus, and more than two-thirds showing gingival bleeding. Second, this clinical burden translates into a measurable impact in everyday life: three-quarters of participants reported at least one Child-OIDP impact, and eating was the domain most often affected. Third, after multivariable adjustment, calculus and caries—not gingival bleeding—were the indicators most consistently associated with poorer Child-OIDP scores. Together, these findings frame oral disease in remote Tibetan adolescents as a public-health problem, not merely a clinical one.

The mean Child-OIDP score of 17.6% of the maximum represents a moderate overall impact on everyday life. Three-quarters of participants reported at least one oral impact, with eating identified as the most affected domain. Other domains—pronunciation, brushing or rinsing, sleeping, smiling, mood and emotions, and attending school—were also affected but to a lesser degree. The pattern is consistent with oral disease that is widespread but typically subclinical in severity, yet still functionally disruptive—particularly for chewing and eating, which are central to overall wellbeing in this age group.

Multivariable regression identified dental calculus as the indicator most strongly associated with higher Child-OIDP scores, with DMFT >0 contributing additionally. Gingival bleeding showed a non-significant negative association; therefore, this finding should be interpreted cautiously and considered hypothesis-generating. Future studies should use formal periodontal probing depth and clinical attachment loss measurements rather than dichotomous bleeding alone. Overall, these results support integrating patient-reported outcomes with clinical assessment to better understand the broader effects of oral disease.

Compared with the recent study by Duan et al., which reported a high prevalence of oral disease in Tibetan early adolescents but did not find a robust correlation with QoL, our caries prevalence is consistent with their findings, and we additionally show significant associations of DMFT and dental calculus with poorer Child-OIDP scores. The discrepancy may reflect our larger sample size and an older mean age (13–15 years vs. their mean of 12 years) (17).

Our prevalence estimates align with those of Campisi et al. (18), who studied a similarly sized cohort (*n* = 916) of children in a Tibetan settlement and reported an oral disease prevalence of roughly 70%, although they did not assess OHRQoL. Guan et al. (19) studied older Tibetan adults (≥35 years) and are cited here only to provide a broader context on oral-health burden in Tibetan populations; their findings are not used as direct comparisons with our adolescent OHRQoL results.

In a larger survey published by Hou et al. (20), the reported prevalence of oral-health problems in Tibetan adolescents was lower (~39%). Still, the authors documented low oral-health awareness and attributed the pattern to the geographic environment, dietary habits, student attitudes, and a lack of oral-health promotion and education (20).

In a systematic review of 23 studies, Alvarez-Azaustre et al. (21) reported that eating is the most frequently affected dimension and toothache the most frequent cause of OHRQoL impact, and that the QoL impact is greater in younger adolescents—patterns that are mirrored in our cohort.

The substantial burden of oral-health problems in our sample may be related to a combination of suboptimal oral-hygiene practices, frequent sugar consumption, and limited access to dental care. Study data indicate that only one in ten participants (9.4%) brushed their teeth twice daily, well below recommended oral-hygiene standards.

In addition, approximately half of participants (52.7%) were unsure whether their toothpaste contained fluoride, suggesting limited awareness or limited availability of preventive dental products. These inadequate practices are consistent with the observed clinical outcomes—almost half of the participants had a DMFT score greater than zero—but the cross-sectional design of our study does not establish causality.

Dietary habits further exacerbate this situation, with nearly half of our cohort participants (40.2%) consuming sweet snacks daily and 27.4% drinking sweet beverages daily. Frequent free-sugar intake is an established risk factor for dental caries, as supported by the WHO guideline on sugar intake and the systematic review by Moynihan and Kelly (22) and World Health Organization (23). In this cross-sectional study, sugar intake should be interpreted as an associated contextual factor rather than a proven cause.

Moreover, the majority of them (77.2%) had never visited a dentist before. This lack of professional care means that many oral-health issues remain untreated, allowing them to worsen over time and compound the disease burden in this population.

Our high Child-OIDP questionnaire results are directly linked to the high prevalence of caries; for example, tooth-specific findings showed that the first mandibular molars (teeth 36 and 46) were crowned in 17.0 and 17.8% of participants, respectively. Such advanced decay or damage often leads to pain, sensitivity, or difficulty chewing, impairing the ability to eat comfortably.

Beyond eating, smiling, and pronunciation were also affected. These impacts may reflect aesthetic and social concerns, which are particularly salient during adolescence—a developmental stage characterized by heightened self-awareness and sensitivity to peer perceptions. Dental calculus showed a strong cross-sectional association with higher Child-OIDP scores. Because of the cross-sectional design, we cannot determine whether calculus and caries cause QoL impairment or whether the three are linked through shared upstream factors such as oral-hygiene behavior, frequency of sugar intake, and lack of professional dental care. We therefore report these as associations rather than causal effects.

Our findings call for early oral-health education, a clinical focus on early caries treatment, and policy efforts to enhance access to dental service in our County.

Our study has several important limitations. First, the cross-sectional design precludes causal inference: any associations reported between clinical oral indicators and Child-OIDP scores represent a temporal snapshot, and reverse causation (for example, that pain-driven QoL impairment delays dental care and therefore increases observed calculus burden) cannot be excluded. Second, unmeasured confounders —including household socioeconomic status, water fluoridation status, and dietary detail beyond sugar frequency—were not captured and could underlie the observed associations. Third, the adolescent sample, although large, was drawn from a single school in a single remote county and may not generalize to other Tibetan or Chinese adolescent populations. Fourth, the adult cohort was small, hospital-based, and recruited by convenience, and should therefore be interpreted only as a descriptive context, not as a representative sample of Tibetan adults. Fifth, the Child-OIDP was administered in Chinese with oral Tibetan clarification rather than as a formally translated and back-translated Tibetan instrument; the future study should undertake full cross-cultural adaptation and psychometric revalidation. Finally, self-reported items, including the frequency of brushing and sugar intake, are subject to recall and social desirability bias.

We recommend that the future studies use longitudinal designs with larger, more geographically diverse samples that include all age groups, formally validated Tibetan-language instruments, and richer measurement of socioeconomic and behavioral confounders, with continued emphasis on patient-reported QoL outcomes alongside clinical indicators.

## 5 Conclusion

The high prevalence of oral-health problems among Tibetan adolescents in Suoxian County occurred alongside suboptimal oral-hygiene practices, frequent sugar consumption, and limited access to dental care. These factors are associated with widespread caries and periodontal findings, which in turn are associated with poorer Child-OIDP scores. The association between dental calculus and OHRQoL impact in particular highlights the public-health relevance of chronic oral conditions in this population, while the weaker effect of gingival bleeding points to differences in severity and perception. These findings—although cross-sectional and therefore not causal — underscore the need for targeted preventive and therapeutic interventions to improve oral health in this remote Tibetan population.

## Data Availability

The raw data supporting the conclusions of this article will be made available by the authors, without undue reservation.

## Footnotes

1
https://www.jamovi.org

## References

[1] Alsumait A ElSalhy M Amin M. Long-term effects of school-based oral health program on oral health knowledge and practices and oral health-related quality of life. Med Princ Pract. (2015) 24:362–8. doi: 10.1159/000430096, 26045154 PMC5588237

[2] Williams DM. The research agenda on oral health inequalities: the IADR-GOHIRA initiative. Med Princ Pract. (2014) 23:52–9. doi: 10.1159/000356934, 24401749 PMC5586947

[3] Kay E Locker D. A systematic review of the effectiveness of health promotion aimed at improving oral health. Community Dent Health. (1998) 15:132–44. 10645682

[4] Yewe-Dyer M. The definition of oral health. Br Dent J. (1993) 174:224–5. doi: 10.1038/sj.bdj.4808131, 8461192

[5] Locker D Quiñonez C. To what extent do oral disorders compromise the quality of life? Community Dent Oral Epidemiol. (2011) 39:3–11. doi: 10.1111/j.1600-0528.2010.00597.x, 21114518

[6] McGrath C Broder H Wilson-Genderson M. Assessing the impact of oral health on the life quality of children: implications for research and practice. Community Dent Oral Epidemiol. (2004) 32:81–5. doi: 10.1111/j.1600-0528.2004.00149.x, 15061856

[7] Sischo L Broder HL. Oral health-related quality of life: what, why, how, and future implications. J Dent Res. (2011) 90:1264–70. doi: 10.1177/0022034511399918, 21422477 PMC3318061

[8] Liu Z McGrath C Hägg U. The impact of malocclusion/orthodontic treatment need on the quality of life. A systematic review. Angle Orthod. (2009) 79:585–91. doi: 10.2319/042108-224.1, 19413386

[9] Piovesan C Antunes JLF Guedes RS Ardenghi TM. Impact of socioeconomic and clinical factors on child oral health-related quality of life (COHRQoL). Qual Life Res. (2010) 19:1359–66. doi: 10.1007/s11136-010-9692-7, 20571918

[10] Porritt JM Rodd HD Baker RS. Quality of life impacts following childhood dento-alveolar trauma. Dent Traumatol. (2011) 27:2–9. doi: 10.1111/j.1600-9657.2010.00943.x, 21129159

[11] Abanto J Carvalho TS Mendes FM Wanderley MT Bönecker M Raggio DP. Impact of oral diseases and disorders on oral health-related quality of life of preschool children. Community Dent Oral Epidemiol. (2011) 39:105–14. doi: 10.1111/j.1600-0528.2010.00580.x, 21029148

[12] Wong HM McGrath CPJ King NM Lo ECM. Oral health-related quality of life in Hong Kong preschool children. Caries Res. (2011) 45:370–6. doi: 10.1159/000330231, 21822015

[13] Barbosa TS Gavião MBD. Oral health-related quality of life in children: part II. Effects of clinical oral health status. A systematic review. Int J Dent Hyg. (2008) 6:100–7. doi: 10.1111/j.1601-5037.2008.00293.x, 18412721

[14] Oral Health Surveys. Basic Methods - 5th edition. (2013). Available online at: https://www.who.int/publications/i/item/9789241548649 (Accessed March 4, 2025).

[15] Gherunpong S Tsakos G Sheiham A. The prevalence and severity of oral impacts on daily performances in Thai primary school children. Health Qual Life Outcomes. (2004) 2:57–7. doi: 10.1186/1477-7525-2-57, 15476561 PMC529463

[16] Yusuf H Gherunpong S Sheiham A Tsakos G. Validation of an English version of the child-OIDP index, an oral health-related quality of life measure for children. Health Qual Life Outcomes. (2006) 4:38–8. doi: 10.1186/1477-7525-4-38, 16813660 PMC1533817

[17] Duan S Tang R Zhang C Su Q Yang H Cai H. The correlation of region-specific lifestyle and subjective perception of oral health with oral health-related quality of life among Tibetan early adolescents in Ganzi: a cross-sectional study. PeerJ. (2025) 13:e18842. doi: 10.7717/peerj.18842 39872029 PMC11771303

[18] Campisi G Buttacavoli F Neri B Capocasale G Mauceri N Mauceri R. Oral health status of 916 children in Tibetan settlement (Bylakuppe, India): a cross-sectional descriptive study. Int J Paediatr Dent. (2024) 34:925–32. doi: 10.1111/ipd.13193, 38659165

[19] Guan L Guo J Ban J Li G Tong J Chuan A. Status of dental caries and associated factors in Tibetan adults: findings from the fourth China national oral health survey. BMC Oral Health. (2020) 20:248–8. doi: 10.1186/s12903-020-01225-0, 32894126 PMC7487806

[20] Hou R Mi Y Xu Q Wu F Ma Y Xue P. Oral health survey and oral health questionnaire for high school students in Tibet, China. Head Face Med. (2014) 10:17–7. doi: 10.1186/1746-160X-10-17, 24884668 PMC4049488

[21] Alvarez-Azaustre MP Greco R Llena C. Oral health-related quality of life in adolescents as measured with the child-OIDP questionnaire: a systematic review. Int J Environ Res Public Health. (2021) 18:12995–5. doi: 10.3390/ijerph182412995, 34948611 PMC8701449

[22] Moynihan PJ Kelly SAM. Effect on caries of restricting sugars intake: systematic review to inform WHO guidelines. J Dent Res. (2014) 93:8–18. doi: 10.1177/0022034513508954, 24323509 PMC3872848

[23] World Health Organization. (2015). Guideline: Sugars Intake for Adults and Children. World Health Organization. Available online at: https://www.who.int/publications/i/item/9789241549028 (Accessed July 28, 2026).

